# Peripheral dose distributions for a linear accelerator equipped with a secondary multileaf collimator and universal wedge

**DOI:** 10.1120/jacmp.v3i4.2554

**Published:** 2002-09-01

**Authors:** Sasa Mutic, Jacqueline Esthappan, Eric E. Klein

**Affiliations:** ^1^ Department of Radiation Oncology Mallinckrodt Institute of Radiology, Washington University School of Medicine St. Louis Missouri 63110

**Keywords:** Elekta, multileaf collimator, universal wedge, peripheral dose

## Abstract

The American Association of Physicists in Medicine Task Group 36 (AAPM TG‐36) data can be used to estimate peripheral dose (PD) distributions outside the primary radiation field. However, the report data does not apply to linear accelerators equipped with a multileaf collimator (MLC) and universal wedge (UW). Tertiary multileaf collimators have been shown to significantly affect PD distributions and TG‐36 reported data. Measurements were performed to evaluate PD distributions for a linear accelerator equipped with a secondary MLC, backup diaphragms, and UW. This data can be used to compliment the TG‐36 report for estimation of doses to critical structures outside primary radiation fields. For the evaluated linear accelerator, an MLC is incorporated in the upper secondary collimator jaws. Backup shielding diaphragms are located underneath the MLC. At the nominal collimator position, the MLC and the backup diaphragm provide collimation primarily in the transverse direction. Conventional, solid tungsten‐alloy jaws, located underneath the backup diaphragms, provide secondary collimation in the longitudinal direction. The universal wedge provides dose modulation in the direction of the conventional jaws. Measurements were made with an ionization chamber inserted into a $20\times 40\times 120\;{\text{cm}}^{3}$ water‐equivalent plastic phantom with the secondary collimator and MLC settings of 5×5,10×10,15×15, and $25\times 25\;{\text{cm}}^{2}$ with and without UW. Data was acquired along the machine's longitudinal axis for 6, 10, and 18 MV photons. Peripheral dose distributions were measured with the collimator rotated to 0^°^ and 270^°^ for open field measurements and to 0^°^, 180^°^, and 270^°^ for wedged fields (IEC 1217). This allowed evaluation of peripheral dose distributions as a function of collimator rotation. Wedged fields were normalized to deliver the same dose at the depth of maximum dose on the central axis as open fields. The measured PD distributions were generally comparable to data reported by TG‐36. At distances close to the field edge (less than 30 or 40 cm), the measured PD distributions were lower when the measurement point was shielded by solid jaws than with MLC and backup diaphragm. At longer distances, this trend reversed for all energies and evaluated field sizes. However, the difference in PD distribution with collimator rotation was not large enough to warrant strategic positioning of the collimator to reduce dose to critical structures outside the primary radiation field. Because internal scatter dominates close to the field edge, wedged PD distributions were comparable to open field doses at distances closer than 30 cm. However, at distances larger than 30 cm from the field edge, wedged PD distributions were significantly grater than those for open fields due to increased contribution of leakage radiation. Increased leakage radiation is due to the increase in wedged field monitor units, which is related to a small wedge factor (0.27 to 0.29).

PACS number(s): 87.53.–j, 87.66.–a

## INTRODUCTION

As we have previously described,^1^ radiation doses to critical structures (e.g., fetus, ovaries, testes, thyroid, pacemaker, and defibrillator) outside the primary radiation field usually must be evaluated prior to treatment and reduced when necessary. The American Association of Physicists in Medicine Task Group 36 (AAPM TG‐36) data^2^ can be used to estimate peripheral dose (PD) distributions for various treatments and to determine the need for additional shielding. However, the report data were obtained on linear accelerators without multileaf collimator (MLC) and universal wedge (UW).

Peripheral dose distributions consist of internal scatter, collimation scatter, transmission through collimation, head leakage, and room scatter. The use of a tertiary MLC has been shown to significantly reduce PD due to a reduction in scatter from the primary and secondary collimator, transmission through the secondary collimator, and head leakage.^1^ Similarly, a linear accelerator equipped with a secondary multileaf collimation and backup shielding diaphragms and UW can produce PD distributions substantially different from those reported by TG‐36. This paper reports peripheral dose distributions for such a linear accelerator. The measured dose distributions can be used to compliment the TG‐36 report data for estimation of doses to critical structures outside primary radiation fields.

## METHODS AND MATERIALS

The measurements were performed on a multimodality linear accelerator (Elekta Precise, Elekta Norcross, GA) equipped with an MLC and universal wedge. The machine delivers photons with nominal energies of 6, 10, and 18 MV. The MLC design and performance characteristics have been described by Jordan and Williams.^3^ The MLC replaces the upper secondary collimator jaw on a conventional linear accelerator and is complemented by backup diaphragms which are located underneath the MLC.

**Figure 1:**
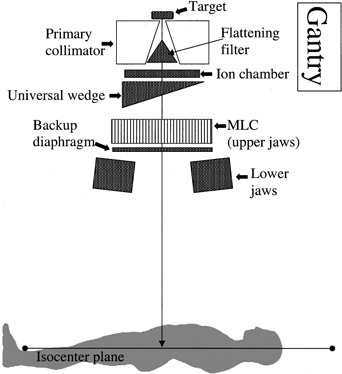
Simplified side view of the gantry head with the collimator at 0° in relationship to the patient lying on the treatment table. When retracted, the MLC collimator is lateral to the patient.

The lower secondary collimation consists of two opposing solid‐tungsten‐alloy jaws and is located inferior to the backup diaphragms. At the nominal collimator and treatment table position of 0° (IEC 1217),^4^ the MLC is oriented perpendicular to the longitudinal axis of a patient lying on the table. Figure 1 shows a simplified schematic of the gantry head, in nominal position, in relationship to a patient on the treatment table. In this situation, the majority of the patient's anatomy outside the primary radiation field is shielded by the lower secondary jaws and partially by the outermost MLCs. When the collimator is rotated by 90° in either direction, the patient is primarily protected by MLCs and backup diaphragms. The UW is a motorized wedge filter that can be positioned in or out of the radiation beam and is located above the MLC (Fig. 1) and provides dose modulation in the direction orthogonal to MLC orientation. The filter is nominally a 60° wedge, and other wedge angles are obtained by a combination of open and wedged fields.^5^ The maximum field size in the wedged direction is 30 cm. With the collimator at the nominal 0° position, the wedge “heel” is located away from the gantry.

Peripheral dose measurements were performed using a 0.6 cm^3^ Farmer‐type ionization chamber (PTW N23333, Friedberg, Germany) inserted into a $20\times 40\times 120{\text{cm}}^{3}$ water‐equivalent plastic phantom (Solid Water model 457, Gammex/RMI, Milwaukee, WI) and connected to a calibrated electrometer (Keithley model 602, CNMC, Nashville, TN.). For all measurements, the ionization chamber was placed at the level of isocenter plane at a depth of 10.0 cm in the phantom (midplane). Due to small PD depth dependence,^1^ ^,^ ^2^ ^,^ ^5^ ^–^ ^7^ PD distributions were evaluated only at that depth. The number of monitor units was adjusted to maintain the ionization reading precision at 1%. Uncertainty in the measured dose due to positional inaccuracy was not considered. Due to the slope of the PD distribution curve, uncertainty due to spatial inaccuracy was greatest at points close to the radiation field and became negligible at points far from the field edge (greater than approximately 40 cm).

Positional inaccuracy was within 2 mm. The output of the linear accelerator was monitored at the beginning and end of each measurement session and was constant within 1%. The long dimension of the phantom placed on the treatment table corresponded to the superior‐inferior axis of a patient lying on the treatment table. Data were taken at distances up to 70 cm away from the primary radiation field edge. For all measurements, both the treatment table and gantry were placed at 0° (IEC 1217).

Data were acquired for 6, 10, and 18 MV photons. These measurements did not account for dose contributions from photoneutrons. As previously discussed^1^ and pointed out in the TG‐36 report, the contribution of neutrons to the total PD is small near the beam edge. At further distances, the total PD is smaller, but the fractional contribution from the photoneutrons can be high.^2^ ^,^ ^8^ The National Council on Radiation Protection^9^ considers the risk of long‐term biological effects of incidental neutrons from the linear accelerator to be negligible.

Peripheral dose measurements were made for open and wedged fields. McParland^5^ has shown that PD distributions can be significantly affected by the UW.

Peripheral dose distributions for open fields were evaluated for 5×5,15×15, and $25\times 25{\text{cm}}^{2}$ secondary collimator and MLC setting and for 0° and 270° collimator rotation. For 0° setting, measurement points were shielded by the lower secondary collimator jaws and the outermost MLC leafs (Fig. 1). MLC and backup diaphragms provided shielding for 270° collimator setting. Collimator rotation has been shown to considerably affect PD distributions.^1^ ^,^ ^5^ ^–^ ^6^

For 60° wedged fields, PD distributions were evaluated for 5×5,10×10, and $15\times 15{\text{cm}}^{2}$ for 0°, 180°, and 270° collimator rotation (270° measurements were not acquired for the $10\times 10{\text{cm}}^{2}$ setting). For the 0° setting, measurement points were inferior to the wedge “heel.” At 180° rotation, the wedge “toe” is located superior to measurement points. With the 270° rotation, measurement points are aligned in the nonwedged direction. The UW has a relatively small wedge factor, which typically ranges from approximately 0.27 to 0.29 as function of beam energy, field size, and depth. The small wedge factor requires a significant increase in monitor units to deliver the same dose on central axis as open fields. Leakage radiation is proportional to monitor units used for treatment delivery. Therefore, it is expected that wedged fields, delivering the same dose on central axis as open fields, will have peripheral doses approximately four times larger than equivalent open fields. All measured PD distributions were normalized to 100% on the central axis at the depth of maximum dose.

## RESULTS

**Figure 2:**
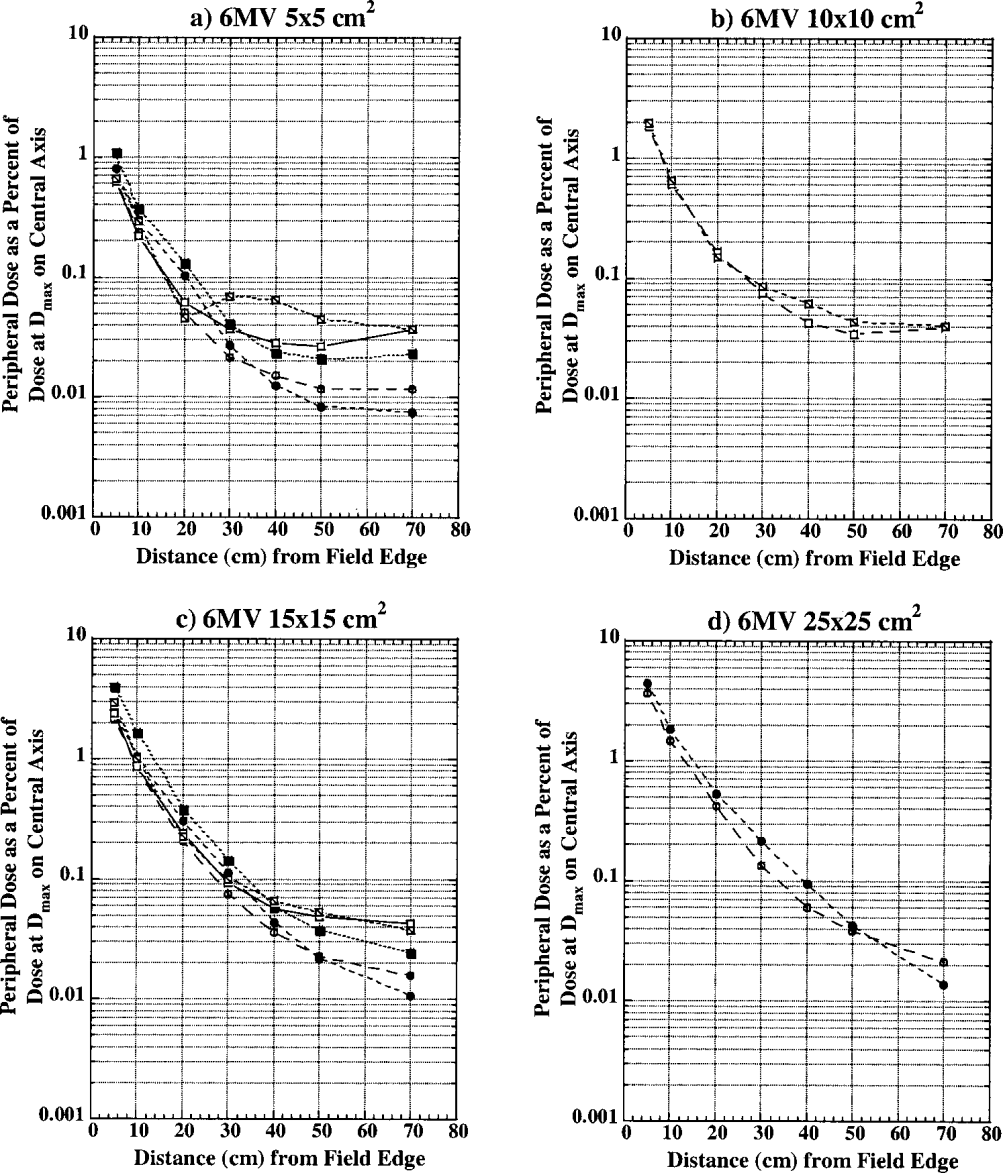
Peripheral dose in phantom from 6 MV photons for field sizes (a) $5\times 5{\text{cm}}^{2}$, (b) $10\times 10{\text{cm}}^{2}$; (c) $15\times 15{\text{cm}}^{2}$; and (d) $25\times 25{\text{cm}}^{2}$ at 10 cm depth, normalized to 100% on the central axis at depth of maximum dose (1.5 cm). Open circles, collimator at 0°, open field; solid circles, collimator at 270°, open field; open squares, collimator at 0°, wedged field; closed squares, collimator at 270°, wedged field; crossed squares, collimator at 180°, wedged field.

**Figure 3:**
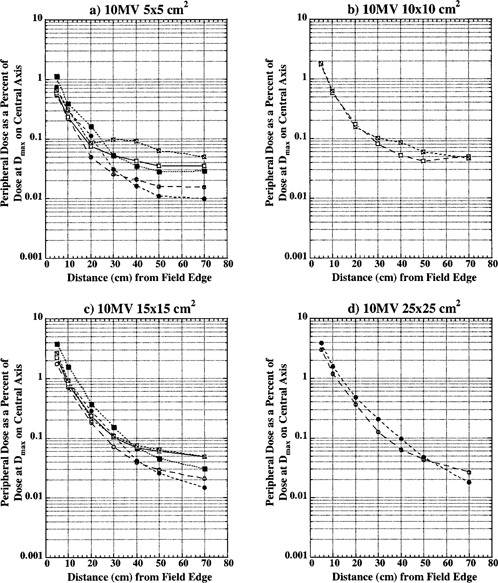
Peripheral dose in phantom from 10 MV photons for field sizes (a) $5\times 5{\text{cm}}^{2}$, (b) $10\times 10{\text{cm}}^{2}$; (c) $15\times 15{\text{cm}}^{2}$; and (d) $25\times 25{\text{cm}}^{2}$ at 10 cm depth, normalized to 100% on the central axis at depth of maximum dose (2.5 cm). Open circles, collimator at 0°, open field; solid circles, collimator at 270°, open field; open squares, collimator at 0°, wedged field; closed squares, collimator at 270°, wedged field; crossed squares, collimator at 180°, wedged field.

**Figure 4:**
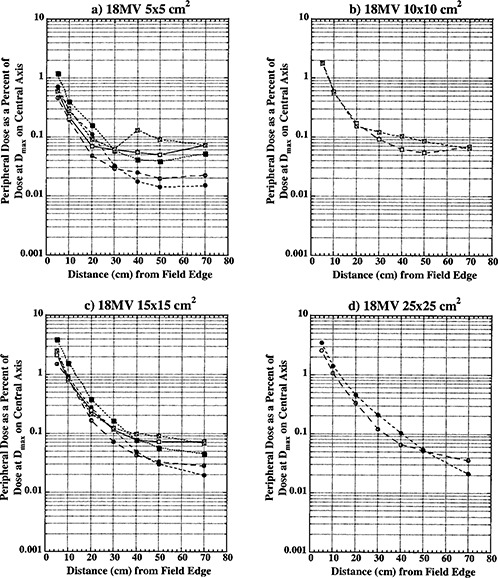
Peripheral dose in phantom from 18 MV photons for field sizes (a) $5\times 5{\text{cm}}^{2}$, (b) $10\times 10{\text{cm}}^{2}$; (c) $15\times 15{\text{cm}}^{2}$; and (d) $25\times 25{\text{cm}}^{2}$ at 10 cm depth, normalized to 100% on the central axis at depth of maximum dose (3.0 cm). Open circles, collimator at 0°, open field; solid circles, collimator at 270°, open field; open squares, collimator at 0°, wedged field; closed squares, collimator at 270°, wedged field; crossed squares, collimator at 180°, wedged field.

Figures 2, 3, and 4 show peripheral dose distributions for 6, 10, and 18 MV photon beams. Each figure contains data for 5×5,10×10,15×15, and $25\times 25{\text{cm}}^{2}$ fields. All data points have 5% error bars applied. Due to marker size and small error magnitude, error bars are predominantly masked within individual markers.

Curves with open circles represent data for open fields with 0° collimator rotation. With this collimator rotation, MLCs and backup diaphragms are lateral to measurement points and these points are predominantly shielded with lower secondary, solid‐tungsten‐alloy jaws. Closed circle curves represent open field data with the collimator at 270° rotation. Data reported for this collimator rotation represents PD distributions under upper secondary jaws (MLCs) and backup diaphragms. All of the open field curves are generally comparable with TG‐36 report data for all three photon energies. Collimator rotation results in a relatively small difference in PD distributions, and there does not seem to be a clear advantage of positioning the collimator to a certain setting to reduce PD.

Open squares represent data for wedged fields with the collimator at 0°. For this setting, measurement points were inferior to the wedge “heel.” Closed squares represent PD data under wedge in the nonwedged direction with the collimator at the 270° setting.

Crossed open squares represent PD distributions inferior to the wedge “toe” with the collimator at 180°. An analysis of these three sets of curves shows that universal wedge PD distributions are a function of collimator rotation, which was also reported by McParland.^5^ For $5\times 5{\text{cm}}^{2}$ wedged data there is a significant difference between “heel” and “toe” PD distributions. The separation of curves occurs approximately 20 to 30 cm from the field edge. This increase in dose was also observed by McParland.^5^ The difference between “heel” and “toe” PD distributions decreases with increase in field size. For a $10\times 10{\text{cm}}^{2}$ field, a small difference is still present and it almost completely dissipates for $15\times 15{\text{cm}}^{2}$ field size. Again, a similar trend was observed by McParland.^5^

Peripheral dose distributions for wedged fields in the nonwedged direction are similar in shape to open field distributions but higher in magnitude. As described earlier, wedged fields were normalized to deliver the same dose at the depth of maximum dose on central axis as open fields. Due to the small wedge factor, this required MUs for wedged fields to be almost four times larger than for equivalent open fields. The leakage radiation component of PD distributions is proportional to MUs. Therefore, it is expected that wedged PD distributions will be higher in magnitude than for open fields. For distances closer than 30 cm from field edge, where internal scatter radiation dominates, wedged PD distributions are comparable in magnitude with open field distributions. At larger distances from the field edge, where leakage radiation dominates, wedged field PD distributions become larger in magnitude approaching the ratio of monitor units required to deliver the same dose with wedged and open fields.

As already described, less than 60° wedged distributions are delivered as a combination of open field and 60° wedged fields. For these fields, the effective PD is calculated as a summation of open field and wedged field contributions according to their respective weights. The effective PD will be between open and wedged field distributions presented in Figs. 2, 3, and 4.

## DISCUSSION

Peripheral dose distributions from a linear accelerator can significantly affect treatment techniques of patients with radiosensitive critical structures, which need to be protected. Premeasured PD distributions can be used in the planning of radiation therapy treatments for such patients and to determine the need for additional shielding.^2^ The AAPM TG‐36 data is often used in these situations. However, TG‐36 data is not necessarily appropriate for linear accelerators equipped with MLCs^1^ and special measurements are needed to evaluate PD distributions from these treatment machines. This paper has presented PD distributions for an Elekta Precise linear accelerator equipped with MLC and UW. This data can be used to complement TG‐36 recommendations. In addition to TG‐36 type data, the data presented here also provides PD distributions as a function of collimator rotation and distributions for universal wedge fields.

The measured data showed that MLC incorporated in the upper secondary collimator with backup diaphragms provide approximately the same amount of shielding as the lower solid‐tungsten‐alloy jaws. The PD distributions for open fields for two collimator rotations are comparable and there does not seem to be a clear advantage in positioning the collimator to a certain setting to reduce dose to critical structures outside the primary radiation field.

Another important observation from measured data is the magnitude of wedge field PD distributions in comparison to open field. The ratio of monitor units required to deliver the same dose at the depth of maximum dose on central axis with UW fields and open fields is almost 4. Intuitively, it would be expected that wedged PD distributions would be approximately four times larger than open field distributions due to an increase in leakage radiation which is related to increased MUs. Furthermore, it is expected that these differences would be observed at longer distances from the field edge, where the leakage radiation dominates.^2^ Data presented here supports this expectation. Wedged PD distributions are comparable to open field distributions at distances less than 30 cm from the field edge, and become almost four times greater at longer distances. This is due to approximately equivalent internal scatter contribution for open and wedged fields. Also, the wedge provides additional shielding for collimator scatter and leakage radiation in comparison to open fields. The effect of this shielding is largest underneath the wedge and close to the field edge and eventually becomes smaller at distances far from the field edge. This is important information when designing treatments for patients with radiosensitive structures (pacemaker, defibrillator, gonads, fetus, etc.) which need to be protected. The data presented here demonstrates that the use of UW with a small wedge factor is not necessarily contraindicated for the treatment of these patients. The dose to critical structures located near the field edge may be comparable to open field doses. However, the whole body dose will be higher for UW fields.
